# Evaluation of a new acrylic-lead shielding device for peripheral dose reduction during cone-beam computed tomography

**DOI:** 10.1259/bjro.20220043

**Published:** 2022-11-28

**Authors:** Hidetoshi Shimizu, Koji Sasaki, Takahiro Aoyama, Tohru Iwata, Tomoki Kitagawa, Takeshi Kodaira

**Affiliations:** 1 Department of Radiation Oncology, Aichi Cancer Center Hospital, 1-1 Kanokoden, Chikusa-ku, Nagoya, Aichi, Japan; 2 Graduate School of Radiological Technology, Gunma Prefectural College of Health Sciences, 323-1 Kamioki, Maebashi, Gunma, Japan; 3 Graduate School of Medicine, Aichi Medical University, 1-1 Yazako-karimata, Nagakute, Aichi, Japan

## Abstract

**Objective:**

To clarify the peripheral dose changes, especially in the eye lens and thyroid gland regions, using an acrylic-lead shield in cone-beam computed tomography (CBCT).

**Methods:**

The acrylic-lead shield consists of system walls and a system mat. The radiophotoluminescence glass dosemeter was set on the eye lens and thyroid gland regions on the RANDO phantom. The system mat was laid under the RANDO phantom ranging from the top of the head to the shoulders, and then, the system walls shielded the phantom’s head. Additionally, the phantom was covered anteriorly with a band that had the same shielding ability as the system mat to cover the thyroid gland region. Protocols for CBCT imaging of the thoracic or pelvic region in clinical practice were used. The measurement was performed with and without the acrylic-lead shield.

**Results:**

The dose to the eye lens region was reduced by 45% using the system wall. Conversely, the dose to the thyroid gland was unchanged. The use of the system mat reduced the dose to the thyroid gland region by 47%, and the dose to the eye lens was reduced by 22%. The dose to the eye lens region decreased to the background level using the system walls and mat.

**Conclusion:**

The newly proposed device using an acrylic-lead shield reduced the peripheral dose in CBCT imaging.

**Advances in knowledge:**

Attention is focused on managing peripheral dose in image-guided radiation therapy. The peripheral dose reduction using the acrylic-lead shield is a new proposal in radiotherapy that has never been studied.

## Introduction

Image-guided radiation therapy (IGRT) is a routine technique in modern external beam radiotherapy essential for monitoring changes in the patients’ anatomical shape and assessing the necessity of offline or online re-treatment planning in addition to the correction of patient or target positioning. Radiation-based imaging guides, such as cone-beam computed tomography (CBCT) and megavoltage computed tomography (MVCT), increase the dose to the normal tissues with routine use.^1,2^ An increased dose in the normal tissues is known to cause secondary cancer and increased side-effect risks to normal tissues.^3–6^ Therefore, the IGRT dose reduction based on as low as reasonably achievable principle is an international concern for their prevention.

Typical methods for IGRT dose reduction have been to optimize imaging parameters (*e.g.,* the number of frames, kVp, and mAs),^7–11^ beam incident direction,^11,12^ and image acquisition field size^12^ for IGRT images as recommended by the American Association of Physicists in Medicine Task Group (AAPM-TG) 180 report.^1^ Other approaches to the IGRT dose reduction have also been reported.^13,14^ The Radiation Oncology Discipline of Children’s Oncology Group proposed a pediatric IGRT modality/frequency decision tree based on the required patient or target positioning alignment quality from the survey of IGRT practice patterns.^13^ Ordóñez-Sanz et al suggested that CBCT imaging protocols be indicated based on the patient’s body size.^14^ In this way, several studies implemented the IGRT dose reduction.^13,14^

This study focused on adjusting the peripheral dose to further reduce the IGRT dose to the normal tissues. The IGRT peripheral dose has been rarely reported. Jia et al measured the peripheral dose from an MV-CBCT system and concluded that the peripheral dose due to MV-CBCT imaging might be significant during the daily application of the MV-CBCT procedure.^15^ Perks et al found doses of >1 cGy per scan outside the CBCT imaged volume.^16^ Apart from the IGRT dose, some studies reported that low radiation dose was associated with the onset of cataracts, thyroid diseases, azoospermia, loss of ovarian function, and oocyte death.^17–22^ Su et al indicated that the population exposed to low-dose-rate environmental radiation throughout their lives were highly at risk of cortical and posterior subcapsular lens opacification. They provided a threshold estimate of 140 mGy (90% CI 110–160 mGy) for cortical lens opacities.^17^ Imaizumi et al evaluated the prevalence of thyroid diseases and their radiation dose responses in atomic bomb survivors and reported that approximately 28% of all solid nodules, 37% of malignant tumors, 31% of benign nodules, and 25% of cysts were associated with radiation exposure at mean and median thyroid radiation doses of 0.449 Sv and 0.087 Sv, respectively.^19^ Accordingly, even a low radiation dose like the IGRT peripheral dose should also be reduced.

This study evaluated the peripheral doses of IGRT in the thoracic and pelvic regions using an acrylic-lead shield at the head and neck regions. The use of shields to reduce the IGRT peripheral dose has never been studied, to our knowledge. This study aimed to clarify the peripheral dose changes, especially in the eye lens and thyroid gland regions, using an acrylic-lead shield.

## Methods and materials

### Acrylic-lead shield

**Figure 1.**
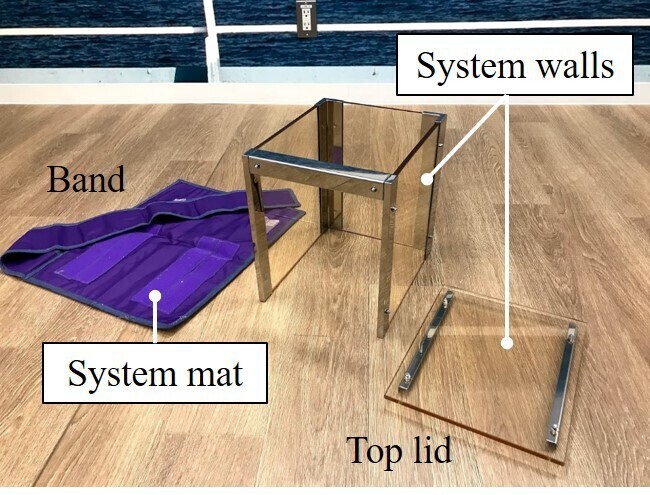
The appearance of the acrylic-lead shield (Pandora box). The Pandora box consists of system walls and a system mat. The system walls are composed of acrylic-lead plates (XA H-8, 0.3 mmPb) with a nominal size of L300 × W250 × H300 mm (top lid: L300 × W250 mm). The system mat is a lead-free material (Bi-layer, 0.5 mmPb) with a nominal size of L485 × W390 mm. A band with the same shielding ability as the system mat extends from the system mat.

Figure 1 shows the appearance of the acrylic-lead shield (Pandora box, Padl, Inc., Japan). The Pandora box consists of system walls and a system mat. The system walls are composed of acrylic-lead plates (XA H-8, 0.3 mmPb) with a nominal size of L300 × W250 × H300 mm (top lid: L300 × W250 mm). The system mat is a lead-free material (Bi-layer, 0.5 mmPb) with a nominal size of L485 × W390 mm. A band with the same shielding ability as the system mat extends from the system mat.

### The dose to eye lens and thyroid regions in thoracic and pelvic CBCT imaging

#### Glass dosemeter calibration

**Figure 2.**
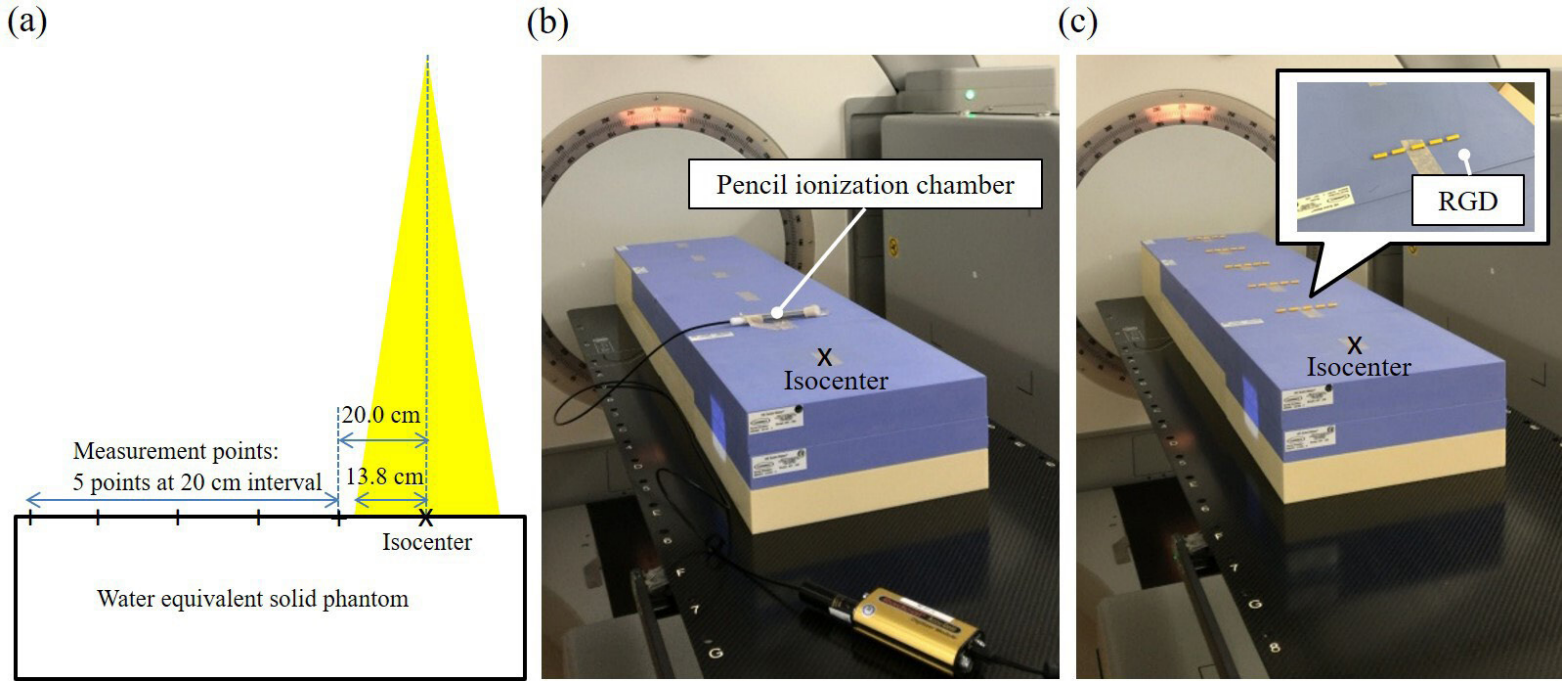
The setup arrangement for the RGD dose calibration. The X-ray tube was placed directly above, and the chamber and RGD were positioned, at the point of 20 cm away from the isocenter to 100 cm away at 20 cm intervals (five points), respectively. RGD; radiophotoluminescence glass dosemeter

The radiophotoluminescence glass dosemeter (RGD, GD-352 M, Asahi Techno Glass, Japan) and an automatic reader (FGD-1000, Asahi Techno Glass, Japan) were used for dose measurement. The GD-352M has a length of 12 mm and a diameter of 1.5 mm with a tin filter in the capsule to lower the energy-dependence effect.^23^ The RGD was calibrated using a pencil ionization chamber (Model 10X6–3 CT, Radcal Inc., Monrovia, CA, USA) with an active length of 100 mm and an active volume of 6 cm^3^. The chamber was connected to a dose monitor (Model 9015, Radcal Inc., Monrovia, CA, USA) for reading. The dosemeter system calibration was traceable by the National Institute of Standards and Technology. Figure 2 shows the setup arrangement of RGD calibration. The X-ray tube (XVI, Elekta Oncology Systems, Crawley, UK) was placed directly above, and the chamber or RGD was placed at 20 cm intervals (five points) from the isocenter to 100 cm as shown in Figure 2b and c, respectively. The RGD calibration curve corresponding to the distance from the field edge at tube voltages of 100 kV and 120 kV was created by dividing the dose obtained from the ionization chamber using the RGD value with background subtraction.

#### Dose measurement in CBCT imaging with and without shield

**Figure 3.**
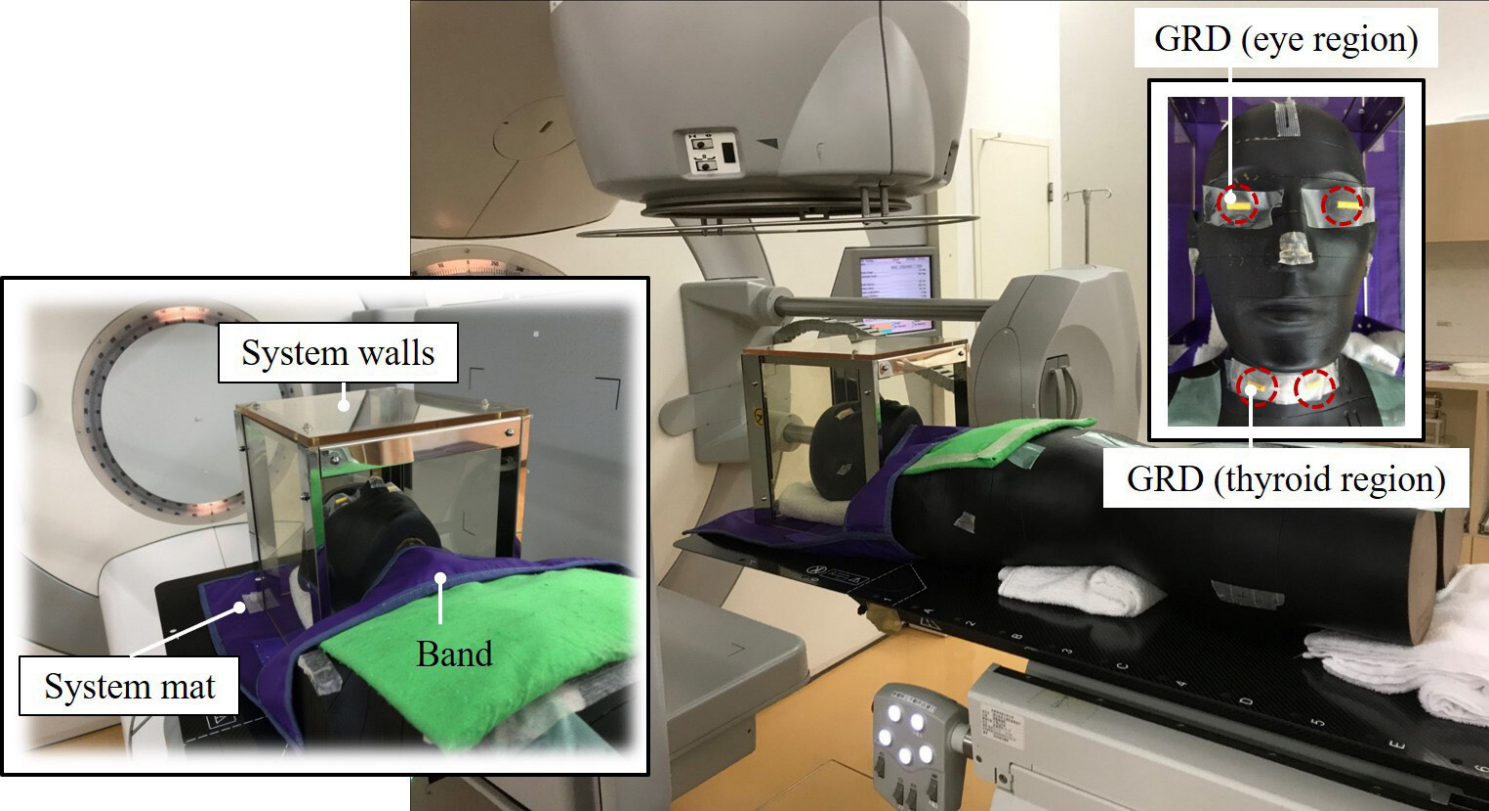
Dose measurement in the CBCT imaging with the shield. Using the RANDO phantom, RGDs were set on the eye lens and thyroid gland regions. A system mat was laid under a RANDO phantom ranging from the head top to shoulders, and then, the system walls shielded the phantom’s head. The bolus with a 5 mm thickness was set on RGDs in the thyroid gland region to express the depth from the patient’s skin surface to the thyroid gland. Additionally, the phantom was covered anteriorly with a band that had the same shielding ability as the system mat to cover the thyroid gland region.

RGDs were set on the eye lens and thyroid gland regions on the RANDO phantom (The Phantom Laboratory, Salem, NY, USA) as shown in Figure 3. The system mat was laid under the RANDO phantom ranging from the head top to shoulders, and then, the system walls shielded the phantom’s head. The bolus with a 5 mm thickness was set on RGDs in the thyroid gland region to express the depth from the patient’s skin surface to the thyroid gland. Additionally, the phantom was covered anteriorly with a band that had the same shielding ability as the system mat to cover the thyroid gland region.

**Figure 4.**
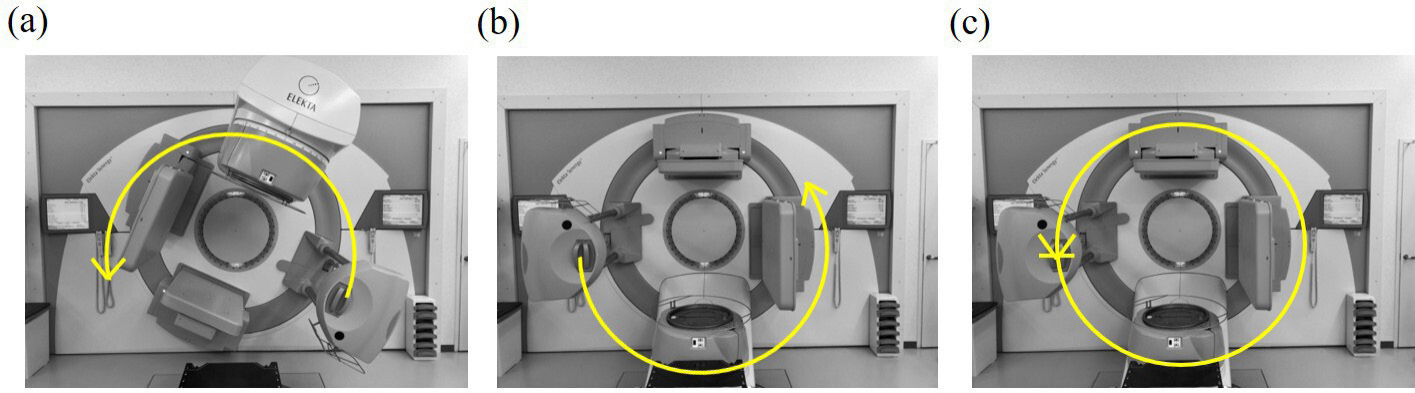
The rotational direction of the X-ray tube in each protocol in Table 2: (**a**) protocol 1, (**b**) protocol 2, and (**c**) protocol 3

Three protocols for CBCT imaging of the thoracic or pelvic region in clinical practice were used. Table 1 shows the protocol details. Only the rotational direction of the X-ray tube to the patient orientation differed between protocols 1 and 2. The rotational direction of the X-ray tube in each protocol is shown in Figure 4 [(a), (b), and (c) for protocols 1, 2, and 3 in Table 1, respectively]. We positioned the RGD at the left and right sides of the eye lens and thyroid gland regions. The measurement was performed twice. Then, the RGD values for each region were averaged after subtracting the background value. Afterward, the RDG value was converted to the dose based on the RGD calibration curve that corresponds to the tube voltage and distance from the field edge in Table 1. The measurement was performed with and without using Pandora box. Only the measurement for protocol one was performed with the system walls or mat to confirm their efficacy.

**Table 1.** Details of the cone-beam CT imaging protocols

	Protocol 1 (For thoracic region)	Protocol 2 (For thoracic region)	Protocol 3 (For pelvic region)
Rotation angle (degrees)	200 (X-ray tube passes in front of the patient)	200 (X-ray tube passes behind the patient)	360
Tube voltage (kV)	100	100	120
mAs	22.0	22.0	234.2
Bowtie filter	On	On	On
Collimator	S20	S20	S10
Collimator opening at 100 cm in the superior-inferior direction (cm)	27.7	27.7	13.5
Distance between the lens or thyroid gland and field edge (cm)	36.2/6.2	36.2/6.2	56.2/46.2

CT, computed tomography.

## Results

### Glass dosemeter calibration

**Figure 5.**
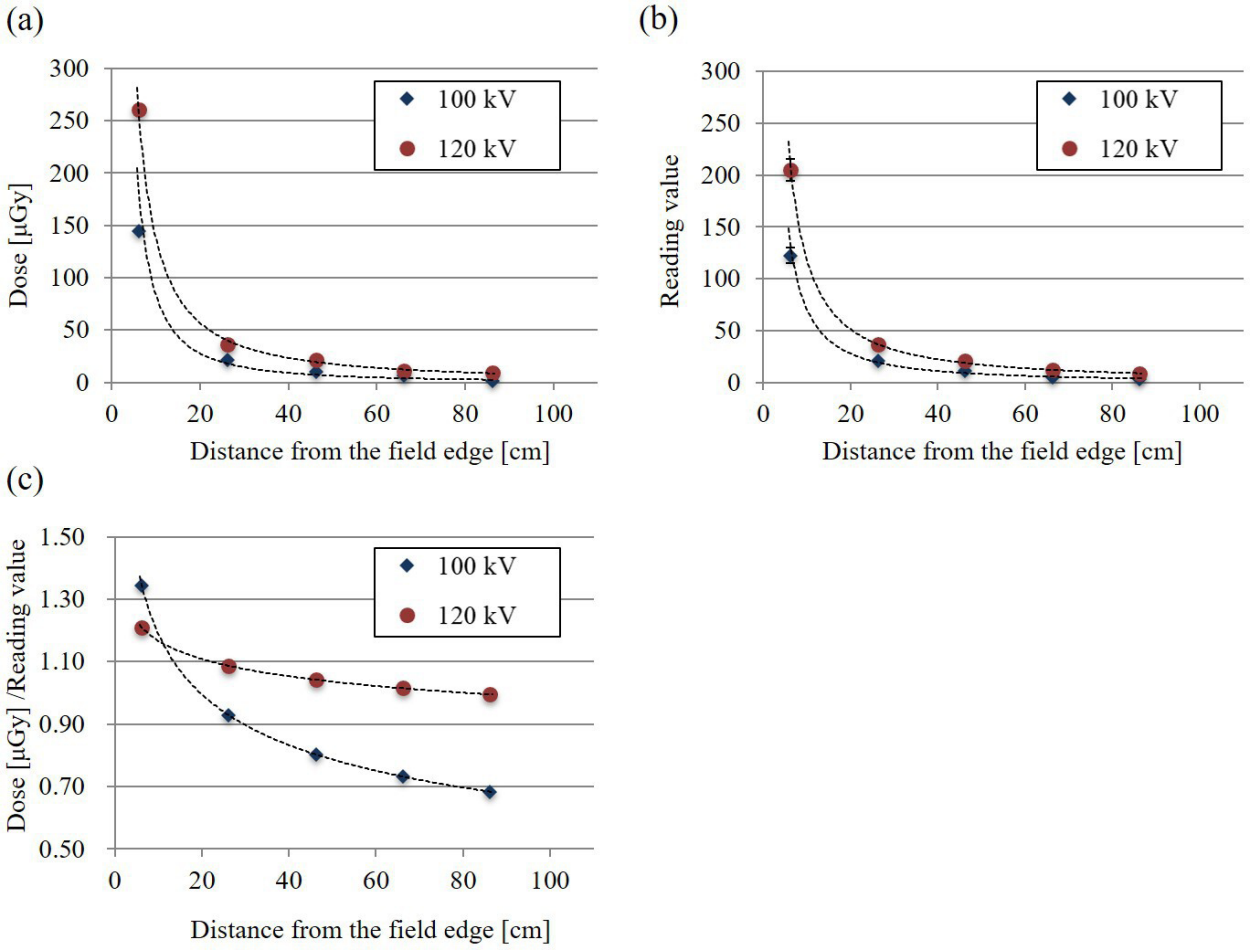
Glass dosemeter calibration. The (**a**) and (**b**) show doses obtained by the pencil ionization chamber and reading values obtained by the RGD, respectively, corresponding to the distance from the field edge of the CBCT. The (**c**) shows the RGD calibration value corresponding to the distance from the field edge. The diamond and circle show the tube voltages of 100 and 120 kV, respectively.

Figure 5a shows the dose obtained using the pencil ionization chamber corresponding to the distance from the field edge of the CBCT with tube voltages of 100 and 120 kV, shown in diamond and circle, respectively. Figure 5b shows the reading values obtained by the RGD corresponding to the distance. Both signals (dose and reading values) decreased as the distance from the field edge increased. The change till the distance of 20 cm from the field edge was large, and the dose was approximately 50 μGy or less at the distance of more than 20 cm from the field edge. Figure 5c shows the RGD calibration value corresponding to the distance from the field edge. The calibration value also decreased with increased distance from the field edge. Further, a calibration curve change of 100 kV concerning the distance was more significant than that of 120 kV.

### Dose measurement in CBCT imaging with and without the shield

Table 2 shows the dose to the eye lens and thyroid gland regions obtained using the RGD in each CBCT imaging protocol. The numbers in parentheses in Table 2 indicate the dose reduction rate when using the shields. When comparing the “system walls only” and “none” in protocol 1, the dose to the eye lens region was reduced by 45% in those using the system wall. Conversely, the dose to the thyroid gland was not changed. When comparing the “system mat only” and “none” in protocol 1, the use of the system mat reduced the dose to the thyroid gland region by 47%. Additionally, the dose to the eye lens was reduced by 22%. When comparing the “Pandora box” and “none” in protocols 1, 2, and 3, the dose to the eye lens region was decreased to the background levels. Further, the doses to the thyroid gland in protocols 1, 2, and three were reduced by 43%, 45%, and 71%, respectively in those using the Pandora box. The system walls or system mat could reduce the dose to the eye lens region or the thyroid gland region, respectively.

**Table 2.** The dose to the eye lens and thyroid gland regions obtained by the RGD in each CBCT imaging protocol. The unit in the figure is μGy

Conditions	Regions	Protocol 1 (For thoracic region)	Protocol 2 (For thoracic region)	Protocol 3 (For pelvic region)
Pandora box (system walls + system mat)	Lens	B.G.	B.G.	B.G.
	Thyroid	29.5 ± 4.8 (43%)	11.7 ± 1.4 (45%)	2.5 ± 1.5 (71%)
System walls only	Lens	3.0 ± 1.2 (45%)	－	－
	Thyroid	53.1 ± 3.6 (0%)	－	－
System mat only	Lens	4.3 ± 0.1 (22%)	－	－
	Thyroid	28.3 ± 0.4 (47%)	－	－
None	Lens	5.5 ± 1.9	1.4 ± 1.7	6.5 ± 0.7
	Thyroid	53.0 ± 1.0	21.1 ± 2.1	8.5 ± 0.5

BG, background; CBCT, cone-beam computed tomography; RGD, radiophotoluminescence glass dosemeter.

The numbers in parentheses indicate the dose reduction rate when using the shields.

## Discussion

In this study, an acrylic-lead shield was used for the head and neck regions to reduce the peripheral dose in thoracic and pelvic CBCT imaging. The acrylic-lead shield, known as the Pandora box, reduced the dose to the eye lens and thyroid gland regions. Significantly, the system walls and mat effectively reduced the dose in the eye lens and thyroid gland regions, respectively.

First, the measurement reliability should be indicated. The RGD used in this study has less fading than the thermoluminescence dosemeter and can be measured repeatedly.^23^ Furthermore, it has been used in radiation protection^24,25^ and dosimetry audit fields.^26,27^ The GD-352M, namely, RGD used in this study, has a tin filter to eliminate overestimated signals in low-energy photons of <100 keV.^28^ Kim reported that measurement uncertainties at the coverage factor of *k* = 2 were 9.26 and 8.16% in dose ranges of 0.005–1 Gy and 1–10 Gy, respectively,^28^ and no large difference was observed between dose ranges. Although their measurement environment differed from that of our research, it sufficiently and accurately evaluated the dose reduction rate using the acrylic-lead shield. According to Perks et al,^16^ the surface dose on a phantom with XVI CBCT imaging was 0.2–0.6 cGy at 6 cm from the CBCT field edge. Conversely, the doses to the eye lens (approximately 6 cm from the field edge) of our thoracic CBCT imaging (protocol 1 and 2) were 5.5 and 1.4 μGy (mean: 3.5 μGy). This dose difference depends on the imaging protocol (Perks et al.: 120 kV, 1040 mAs; our study: 100 kV, 22 mAs). The dose from the linear correction of the mAs value would be evaluated as 0.17 cGy (= 3.5 μGy × 22 mAs/1040 mAs). In Figure 5, although the data were obtained by irradiation directly above (not from rotation-like CBCT), the dose ratio of 120–100 kV at 6 cm from the field edge is approximately doubled, and the corrected dose would be 0.34 cGy. Consequently, if the imaging protocol difference is calculated and compared, the dose obtained in our study would be as close as those of Perks et al. Thus, the measurement reliability in our study is guaranteed.

This study was the first approach using shields to reduce the peripheral dose during radiation-based image-guided radiotherapy and clarified that the dose to the eye lens and thyroid gland regions could be reduced from approximately 50% to the background level using the acrylic-lead shields on the head during thoracic and pelvic CBCT imaging. The AAPM-TG 180 report stated that imaging parameters, beam incident direction, and field size optimization are required to obtain IGRT images; however, it did not mention the application of the shield. This approach, *i.e.,* using the shield, is a newly proposed device in the field of radiotherapy. Applying the shield without hindrance to radiotherapy is effective for further reducing the IGRT peripheral dose.

The obtained peripheral dose in this study was at the µGy level, which was fairly low. However, depending on the IGRT protocol, cumulative peripheral doses from the IGRT during the treatment course could be closer to the treatment dose per fraction (2 Gy). Perks et al reported that peripheral doses of >1 cGy per scan were found outside the CBCT imaged volume.^16^ Furthermore, the peripheral dose apparently increases for the treatment dose; even at 10 cm from the field edge, the peripheral dose was approximately 1% of the central axis dose.^16^ The prescribed dose range for thoracic and pelvic cancers is generally 50–80 Gy, corresponding to 50–80 mGy on the peripheral dose. Partly due to the coronavirus disease pandemic in 2019, hypofractionated schedules have been adopted to reduce the number of visits to the facility and consider the time between surgery and radiotherapy.^29^ Thus, an increase in the treatment dose per fraction (dose rate) would also increase the peripheral dose per fraction. Remarkably, our shield approach can also be applied during treatment and the initial planning CT, not only to the head but also to the thoracic and pelvic regions, although it is necessary to be careful of interference with the patient or affect immobilization in addition to patient discomfort or complaints. If the shielding position can be set for the thoracic and pelvic regions, the shield approach would become more versatile in reducing the peripheral dose.

This study has some limitations. First, the number of measurements by the RGD was small. Therefore, an increased number of measurements would improve the statistical accuracy of the obtained results. Next, the dose was not evaluated separately for the left and right eye lenses. The dose to the left and right structures would differ depending on the direction of rotation of the X-ray tube. In this study, since the primary purpose was to clarify the dose changes in the eye lens and thyroid gland regions using an acrylic-lead shield, dose changes on the left and right were not mentioned.

## Conclusion

This study is the first report of using an acrylic-lead shield to reduce the peripheral dose during CBCT imaging. We clarified that the dose to the eye lens and thyroid gland regions could be reduced from approximately 50% to the background level using the acrylic-lead shields on the head during thoracic and pelvic CBCT imaging. This new approach is expected to reduce the peripheral dose for not only the eye lens and thyroid gland regions but also other parts or not only radiation-based imaging, such as CBCT but also treatment and initial planning CT.
